# 3D bioprinting of skin equivalents: Towards functional wound healing models

**DOI:** 10.1177/20417314251407023

**Published:** 2026-03-30

**Authors:** Adrian Perez-Barreto, Roxana Moscalu, Carlo Tremolada, Marco Domingos, Adam Reid, Jason Wong

**Affiliations:** 1 Blond McIndoe Laboratories, Division of Cell Matrix Biology and Regenerative Medicine, Faculty of Biology, Medicine, and Health, The University of Manchester, UK; 2 Henry Royce Institute, The University of Manchester, UK; 3 Image Regenerative Clinic, Milan, Italy; 4 Department of Mechanical and Aerospace Engineering, School of Engineering, Faculty of Science and Engineering, The University of Manchester, UK; 5 Department of Plastic Surgery & Burns, Wythenshawe Hospital, Manchester University NHS Foundation Trust, Manchester Academic Health Science Centre, UK

**Keywords:** biofabrication, bioprinting, tissue engineering, biomaterials, regenerative medicine, skin models

## Abstract

Developing physiologically relevant human skin models remains a critical challenge in regenerative medicine and disease modelling, particularly for chronic wounds that involve persistent inflammation and vascular dysfunction. Recent advances in 3D bioprinting provide improved spatial organisation and reproducibility compared with other engineering strategies, enabling the fabrication of skin equivalents with increasing structural and cellular complexity. Nevertheless, most current models do not capture the dynamic crosstalk between immune and vascular systems, which is central to wound healing and tissue homoeostasis. This review surveys recent progress in engineering skin models that incorporate immune and vascular elements, and discusses the biological and technological barriers that continue to limit their integration. We also highlight emerging strategies, including organoids, 4D bioprinting, and computational approaches, that may enable next-generation platforms. By more accurately modelling the wound microenvironment, such advances could accelerate translation from laboratory innovation to clinical application.

## Graphical abstract

**Graphical abstract:**
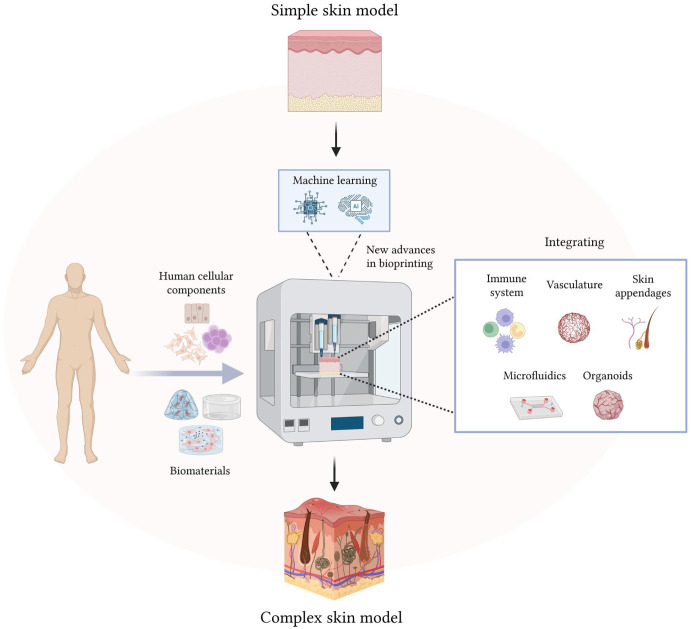

## Introduction

The skin is the largest organ in the human body, playing an important role as a physical barrier and immune defence. As a first-line protection, skin integrity can be frequently affected by numerous external factors, such as heat, mechanical stressors, chemicals, pathogenic microorganisms, and patient comorbidities.^1^ Typically, a small wound in a healthy individual heals within a few days through re-epithelialisation. However, the healing of wounds when there is an extensive loss of skin tissue is more challenging, leading to complex wounds in some cases.^2^ These wounds are characterised by delayed or impaired healing due to a combination of factors such as chronic inflammation, poor vascularisation, and persistent colonisation or even infection.^3^ Moreover, complex wounds are commonly associated with underlying conditions like diabetes, vascular disease, or immune dysfunction. They compromise both the structure and function of the skin and can lead to complications such as amputations and even death in severe cases.^4^ Approximately 1%–2% of the population worldwide will experience a complex wound in their lifetime, with an estimated cost per year to the health service in the UK of over £8 billion, and more than £25 billion worldwide by 2029.^5^ Current therapies, such as autografts, allografts, and commercial skin substitutes, often fall short in promoting complete regeneration due to a poor understanding of the cellular and molecular mechanisms that govern skin regeneration in complex wound environments.^6^ To understand these limitations, in vitro and in vivo models have been produced. However, three-dimensional in vitro models lack the architectural complexity and cellular diversity of native skin, while current preclinical animal models, although more physiologically relevant, still lack accuracy in recapitulating the human-specific cellular interactions and wound environment, limiting their predictive power for therapeutic screening.^7^ Moreover, the shift towards non-animal testing, driven by legislative changes such as European Regulation 1223/2009 and the United States Federal Food, Drug, and Cosmetic Act (2022), along with the 3Rs principle (Reduction, Refinement, and Replacement) in animal experimentation, has accelerated the development of commercial in vitro human skin equivalents (HSEs).^8^ Therefore, there is a critical need for the development of better human skin models to elucidate the mechanisms underlying normal wound healing and to uncover why this process fails in complex wounds.

Biofabrication approaches, especially bioprinting, provide a powerful opportunity to address this need by creating HSEs that closely mimic the structural organisation, functional layers, and dynamic microenvironment of native skin.^9^ The three-layered structure of the skin, composed of the epidermis, dermis, and hypodermis, creates a microenvironment that is challenging to replicate in vitro. Traditional two-dimensional culture systems fail to recapitulate the three-dimensional cellular architecture and extracellular matrix (ECM) interactions crucial for skin function and regeneration.^10^ In this context, bioprinting emerges as a transformative technology that enables the precise spatial deposition of multiple cell types and biomaterials to fabricate three-dimensional, physiologically relevant skin constructs.^11^ By incorporating native-like ECM components and layering distinct skin cell populations, bioprinted skin models hold great promise for advancing wound-healing research and skin regeneration. HSEs can be developed through biofabrication using two main strategies: top-down or bottom-up tissue engineering. In the top-down approach, a pre-formed scaffold is used as a “bed,” and then cells are seeded onto or into it to reconstruct tissue architecture.^12^ However, low cell density and uneven cell distribution resulting from the direct seeding of cells make cellular expansion difficult.^13^ In contrast, in the bottom-up approach, cell-laden building blocks ensure the even distribution of cells, thus increasing the cellular viability and promoting the formation of artificial tissues. Top-down skin tissue engineering remains a viable approach for developing 3D skin models. However, the potential to automate and rapidly scale up these models through bioprinting has shifted increasing interest towards bottom-up technologies in the field of skin engineering.

Bioprinting is a technique that enables the precise and controlled spatial deposition of living cells, biomaterials, and bioactive molecules to create complex, 3D biological tissue constructs. These tissue constructs or models are capable of mimicking the structural and functional organisation of native tissues, including skin.^14,15^ Despite significant advances in 3D bioprinting technologies, most constructs consist of only epidermal and dermal layers, lacking key components such as a perfusable vascular network, immune cells, nerve endings, melanocytes, and skin appendages like hair follicles and sweat glands. This structural incompleteness compromises physiological relevance, particularly in applications requiring long-term viability, such as wound healing or complex wound modelling. While there is extensive literature on bioprinted skin models and biomaterials.^16,17^ To our knowledge, no current review comprehensively addresses how these models can be advanced through the integration of additional physiological features, such as immune components and vascular networks. This review highlights recent developments in biofabricated skin models and their in vivo applications, and discusses how key aspects of wound healing, including immune responses and vascularisation, can be effectively integrated to better mimic native skin and its response to injury.

## Skin structure and non-healing process

The skin is one of the most multifunctional organs in the human body, serving as the primary interface between the internal environment and the external world. It provides essential protective functions against mechanical injury, microbial invasion, ultraviolet radiation, and water loss, while also playing vital roles in thermoregulation, immune defence, and sensory perception.^18^ Skin architecture consists of three distinct but interdependent layers: the epidermis, dermis, and hypodermis. The epidermis is the outermost skin layer, composed of stratified squamous epithelium. Its primary role is to act as a protective barrier, maintained through continuous renewal. This dynamic process ensures resilience against environmental insults while preserving hydration and immune defence.^19^ Beneath it, the dermis consists of fibroblast-rich connective tissue that provides structural and biochemical support through an extracellular matrix of collagen, elastin, and other components, while also housing blood vessels, nerves, hair follicles, and glands.^20^ The deepest layer, the hypodermis, is mainly adipose tissue, offering insulation, cushioning, and energy storage, along with larger blood vessels, lymphatics, and nerves that supply the skin above.^21^

When the skin barrier is breached due to injury, the body initiates a complex cascade to repair and reinforce the affected area, restoring integrity, and preventing further damage.^22^ This process, known as wound healing, is a dynamic process that involves the coordinated interaction of various cellular and molecular components across four overlapping phases: haemostasis, inflammation, proliferation, and remodelling. Briefly explained, haemostasis establishes the fibrin provisional wound matrix, and platelets arrive at the wound, providing initial release of cytokines and growth factors for cell migration, proliferation, and eventually, wound closure.^23^ While haemostasis is taking place, the inflammation phase is initiated. This phase is mediated by white blood cells such as neutrophils, macrophages, and lymphocytes, which remove bacteria and denatured matrix components that slow down healing.^24^ These cells are the second source of growth factors and cytokines. Prolonged, elevated inflammation due to these cells delays wound closure by increasing protease and reactive oxygen species (ROS) levels, which can impair healing.^25^ After inflammation, proliferation starts. In this phase, fibroblasts and myofibroblasts, supported by new capillaries, proliferate and synthesise disorganised ECM and granulation tissue. Basal epithelial cells proliferate and migrate over the granulation tissue to close the wound surface.^26^ Finally, during the remodelling stage, fibroblast and capillary density decrease, and initial scar tissue is removed and replaced by ECM resembling normal skin.^27^

**Figure 1.**
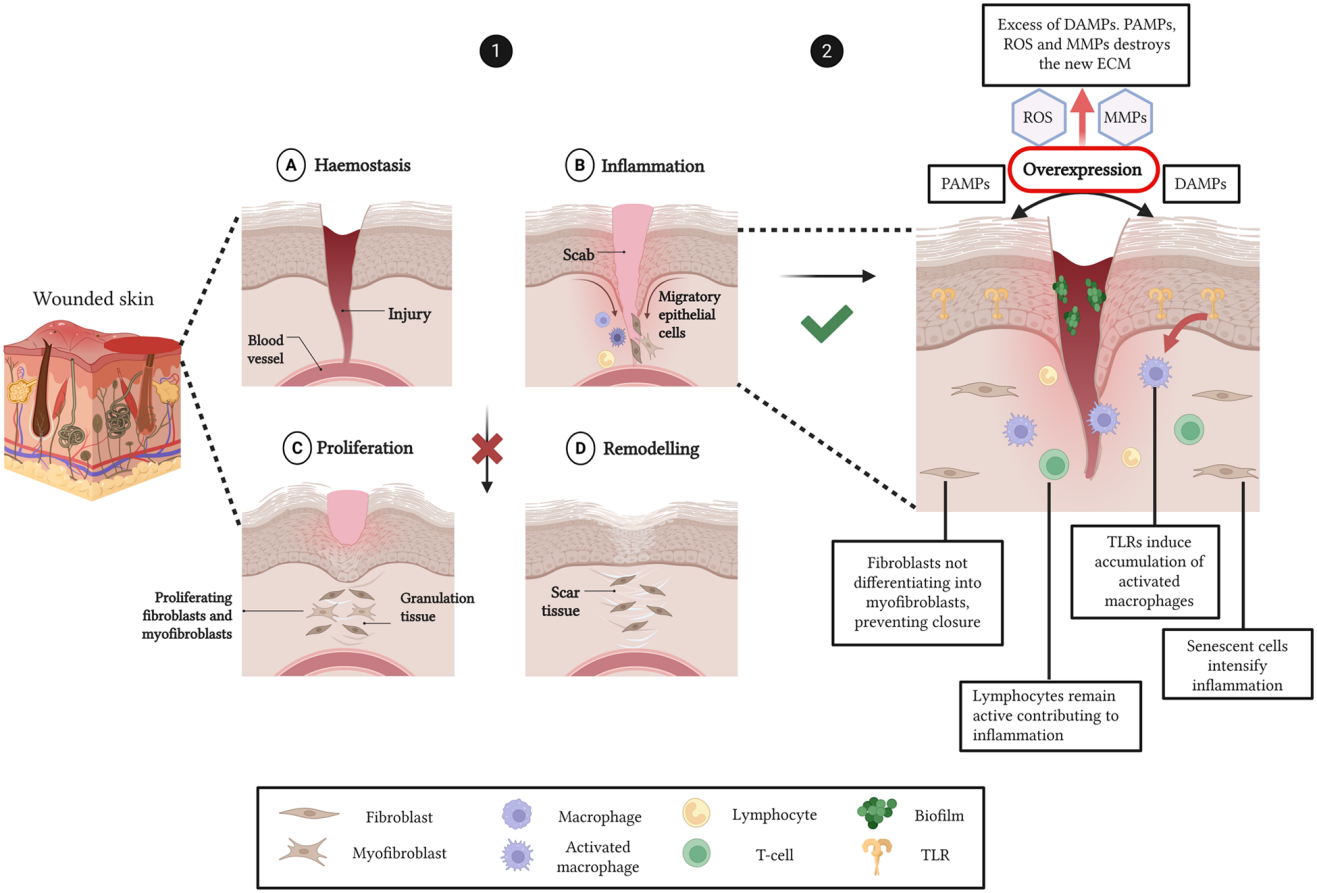
Schematic representation of normal and impaired wound healing pathways. In healthy wound healing (1), tissue injury triggers a coordinated sequence of phases: (a) Haemostasis initiates clot formation and vasoconstriction to prevent blood loss. (b) The inflammatory phase follows, with recruitment of neutrophils and macrophages via DAMPs. Neutrophils clear debris and pathogens, while macrophages regulate the transition to repair by releasing cytokines and growth factors. (c) The proliferative phase involves angiogenesis, fibroblast activation, granulation tissue formation, and re-epithelialisation. (d) The final remodelling phase restores ECM architecture and tissue strength. In contrast, complex wounds (2) fail to progress beyond the inflammatory phase. Sustained immune activation, marked by persistent ROS, MMPs, and pro-inflammatory cytokines, leads to ECM degradation, impaired angiogenesis, and dysfunctional keratinocyte and fibroblast activity. This dysregulated environment prevents resolution of inflammation, thereby stalling regeneration and perpetuating non-healing wound pathology. Created with Biorender.

This is a fine, regulated process that is followed for most of the wounds. However, complex wounds normally do not follow this mechanism, and although they have some differences at the molecular level, they share certain features, including excessive levels of proinflammatory cytokines, proteases, ROS, senescence, persistent infection, and a deficiency of stem cells that are usually dysfunctional (Figure 1).^28,29^ One of the most common characteristics is prolonged inflammation due to repeated tissue injury and infection caused by biofilm.^30^ This inflammation will increase protease levels, such as matrix metalloproteinases (MMPs) that are constantly breaking down the new ECM that is being formed due to damage and pathogen-associated molecular patterns (DAMPs, PAMPs), and dysregulation between MMPs and their inhibitors, toll-like receptors (TLR).^31,32^ Continuous ECM degradation generates bioactive fragments that further amplify inflammation and cellular senescence, perpetuating an “endless” inflammation cycle. Consequently, delayed healing and sustained inflammation exacerbate scar formation and increase the risk of fibrotic complications.^33^

The limited understanding of this non-healing phase has driven the development of HSEs. Unravelling the key mechanisms underlying these complex wounds and having reliable models to test therapeutic strategies will not only facilitate the discovery of new treatments for chronic wounds, but also provide valuable insights into other skin inflammatory conditions.

## Approaches in tissue engineering for skin modelling

### HSEs available in the market

The market for tissue-engineered skin models has expanded significantly over recent decades, driven by advances in biomaterials, cell culture technologies, and bioprinting strategies. Commercially available HSEs can broadly be categorised according to their cellular composition and structural complexity (Table 1). The simplest constructs are epithelial sheets, which replicate only the epidermal layer and are primarily used for studying re-epithelialisation, drug permeation, and epidermal toxicity responses.^34^ While valuable for high-throughput screening, their lack of dermal components limits their physiological relevance, particularly in modelling inflammation or long-term wound closure. Introducing fibroblasts within a matrix gives rise to dermal equivalents, enabling studies of ECM remodelling, growth factor secretion, and fibroblast–keratinocyte crosstalk.^35^ However, these models still lack the complex architecture necessary to capture full skin function. Full-thickness skin equivalents have been developed to incorporate both epidermal and dermal compartments to more closely mimic native skin physiology. These bilaminar constructs are increasingly used to investigate percutaneous absorption, chronic wound repair, and microbial interactions.^36^

**Table 1.** Classification of the different commercial skin models.

Skin models in the market			
Epithelial sheets			
Company	Cell types	Material	References
EpiSkin (Episkin, Lyon, France)	HEK	Bovine collagen matrix and human collagen	Roguet et al.^37^
EpiCS (Phenion, Germany)	HEK	Polycarbonate membrane	Desprez et al.^38^
SkinEthic (Martin Rosdy Laboratories, Nice, France)	HEK	Polycarbonate filter	Pellevoisin et al.^39^
EpiDerm (MatTek Corporation, Ashland, USA)	HEK	Collagen-coated polycarbonate membrane	Netzlaff et al.^40^
ZenSkin (Zen-Bio,USA)	HEK	Polycarbonate filter	Shields et al.^41^
LabCyte EPI-MODEL (J-Tec, Japan)	HEK	Not specified	Kojima et al.^42^
KeraSkin (Biosolution, South Korea)	HEK	Not specified	Jung et al.^43^
Full-thickness skin			
EpiDermFT (MatTek Corporation, USA)	HEK, NHDF	Collagen (No specified)	Hayden et al.^44^
StrataTest (Stratatech, Mallinckrodt Pharmaceuticals, USA)	HEK, NHDF	Type I collagen (Not specified)	Rasmussen et al.^45^
T-Skin (Episkin, France)	HEK, NHDF	Polycarbonate filter	Bataillon et al.^46^
Phenion (Henkel, Germany)	HEK, NHDF	ECM proteins	Jennen et al.^47^
ReproSkin (REPROCELL, Japan)	HEK, NHDF	Alvetex Scaffold	REPROCELL^48^

Current models still fall short of fully replicating the structural and functional complexity of native human skin. Many of these commercial models consist only of partial-thickness constructs, lack appendages such as hair and sweat glands. Furthermore, vascular and nervous networks are absent and fail to reproduce the dynamic interactions between dermal and epidermal layers accurately.^49^ These limitations constrain their use in studying wound healing. As a result, there is growing interest in advancing skin biofabrication, which can more faithfully recapitulate native tissue architecture and physiology. Advancing biomaterial strategies play a critical role in recapitulating the native skin architecture, as they provide structural support and bioactive cues that influence cellular behaviour and dynamic interactions.^50^

### Biomaterial strategies for advancing current HSEs

The mechanical behaviour of human skin plays a fundamental role in wound healing and must be considered when designing physiologically relevant skin models. Native skin exhibits nonlinear, anisotropic, and viscoelastic properties, with elastic moduli typically ranging from 0.05 to 0.5 MPa depending on anatomical site, hydration, and age.^51,52^ This mechanical complexity arises from the hierarchical structure of the dermal ECM, where collagen and elastin fibres provide tensile strength and resilience. Cells interpret these mechanical cues via mechanotransduction pathways, allowing fibroblasts, keratinocytes, and immune cells to modulate proliferation, differentiation, and ECM deposition in response to substrate stiffness and strain.^53,54^ Excessive mechanical stress can lead to delayed healing or hypertrophic scarring, while insufficient tension impairs re-epithelialisation.^55–57^

Recent studies have elucidated specific mechanotransduction mechanisms relevant to skin pathology and regeneration. He et al. demonstrated that increased dermal stiffness drives fibrosis through a Piezo1–Wnt2/Wnt11–CCL24 positive feedback loop, linking substrate rigidity to fibroblast activation and ECM overproduction, highlighting how mechanical cues directly influence skin remodelling and pathological scarring.^58^ Complementing this, Kaiser et al. used bioimaging and RNA-seq to show that dynamic mechanical stimulation modulates fibroblast and keratinocyte gene expression, ECM deposition, and tissue maturation in skin equivalents.^59^ Together, these findings highlight how both static and dynamic mechanical cues can shape tissue structure and function, underscoring the importance of incorporating mechanotransduction considerations into biomaterial and scaffold design.

Given the central role of mechanical cues in skin homoeostasis and repair, these insights directly inform the design of biomaterials for HSEs, which must replicate not only the biochemical cues of the native ECM but also its mechanical and structural properties. Moreover, these materials should allow components such as oxygen, nutrients, or proteins to pass through and provide the cells with all the requirements for proliferation and survival.^60^ A range of different biomaterials has been developed to mimic these mechanical features, providing a supportive matrix that modulates fibroblast proliferation, keratinocyte stratification, and angiogenesis. Fine-tuning parameters such as crosslinking density, degradation rate, and elasticity enables the creation of constructs that maintain mechanical integrity while supporting dynamic cellular processes essential for wound healing. In this way, understanding skin biomechanics directly informs the rational design of bioengineered matrices that can restore both the form and function of native tissue.

HSEs are commonly constructed by embedding fibroblasts within a hydrogel-based scaffold, from either synthetic^61^ or a natural source^62^ to form the dermal layer. Compared with synthetic hydrogels, natural hydrogels offer superior cytocompatibility and contain amino acid sequences that support cellular adhesion, proliferation, and differentiation.^63^ Additionally, natural hydrogels are easily degraded by cellular matrix proteinases, hence, the laden cells can migrate and proliferate. Although many materials are used for skin replacement, especially for wound healing,^64–66^ this review will focus on those commonly used for HSEs development (Table 2).

**Table 2.** Different materials that can be used to build an HSE, with their advantages and limitations.

Materials	Advantages	Downsides	Crosslinking	Reference
Collagen	-High Porosity -Biocompatibility -Enhance cell attachment and proliferation -Absorbability -Improve angiogenesis	-Low solubility and viscosity -Low mechanical properties -Slow gelation time -Formation of fibrotic tissue -The nozzle can be clogged easily	Chemical, physical, and enzyme-induced	Shoulders and Raines^67^, Zhang et al.^68^
Gelatine, gelatine-derived polymers (GelMa)	-Low cost -Biodegradability -Low cytotoxicity -Improve cell adhesion	-Poor mechanical strength -Depends on the temperature -Modification required	Chemical, enzymatic, and physical UV exposure (GelMa-physical)	Hoque et al.^69^, Lukin et al.^70^
Alginate	-Fast gelation -Low cost -Biocompatibility and biodegradability	-Low mechanical strength -Poor cell attachment	Physical	Paques et al.^71^, Zahid et al.^72^
Silk	-High mechanical strength -Printability and high resolution -Controllable degradation -Low immunogenicity -Maintain cell viability	-Poor solubility -Mix with other polymers for optimal rheology and printability -Swelling behaviour -Easily clogs the nozzle	Chemical, physical, and enzyme-induced	Alkazemi et al.^73^, Wang et al.^74^
Fibrinogen	-Biocompatibility -Enhance cell adhesion -Non-cytotoxic -Blood clot formation	-Low mechanical properties -Rapid degradation	Chemical, physical, and enzyme-induced	Anitua et al.^75^, Cavallo et al.^76^
dECM	-Tissue-specific biochemical cues -Mimics the native ECM of the source tissue -Promotes adhesion, proliferation, and differentiation -Contains angiogenic factors that support vascular network formation	-Cytotoxicity if not well processed -Poor mechanical properties -Immunogenicity	Enzymatic or physical	Wang et al.^77^, Mobaraki et al.^78^

Protein-based hydrogels form the cornerstone of biofabrication strategies for human skin equivalents, owing to their biochemical resemblance to the native ECM. Collagen type 1 is the most utilised as it forms the backbone of most native skin ECM, supporting critical biological cues that support cell adhesion, migration, and proliferation, particularly for dermal fibroblasts and keratinocytes.^79^ Moreover, collagen-based hydrogels are highly biocompatible and bioactive, promoting tissue remodelling and integration. Its printability is limited due to low viscosity and weak mechanical properties in its native form.^80^ To improve stability, it is often used at high concentrations or combined with other polymers. Thermal, pH-induced, or enzymatic crosslinking is used to enhance its gelation and structural fidelity post-printing. In bioprinted skin models, collagen-based bioinks are typically used to recreate the dermal compartment, often supporting fibroblasts and endothelial cells.^81^ Recently, Yang et al. developed a recombinant human type III collagen (rhCol3)–GelMA bioink for 3D bioprinting of full-thickness human skin equivalents. The bioink maintained suitable printability and cell viability despite stiffness changes. In vivo, rhCol3-containing constructs enhanced wound closure in a rat model. This study demonstrates that recombinant collagen can improve both the biological functionality and regenerative potential of bioprinted skin while reducing immunogenic risk relative to animal-derived collagen.^82^ Despite its advantages, slow gelation and poor printability remain technical challenges that require formulation optimisation.

To overcome these problems, gelatine has emerged as a possible substitute. Gelatine is derived from the partial hydrolysis of collagen and retains many integrin-binding motifs (such as RGD sequences) that promote cell adhesion. Moreover, it is thermoresponsive, gelatinous at low temperatures and liquefying at physiological temperatures, making it useful in extrusion-based bioprinting. However, unmodified gelatine lacks mechanical strength and long-term stability at body temperature.^83,84^ To address this, gelatine methacryloyl (GelMA) has emerged as a versatile derivative, functionalised with methacrylate groups that allow covalent crosslinking upon UV or visible light exposure in the presence of a photoinitiator. This results in a hydrogel with tuneable stiffness, degradation rate, and improved structural integrity, ideal for high-resolution bioprinting.^85^ GelMA is highly favoured in skin bioprinting due to its cell compatibility, customisability, and reproducible printing behaviour. Pazhouhnia et al. developed a 3D-bioprinted GelMA/gelatin scaffold incorporating amniotic membrane extract (AME) and loaded with keratinocytes, fibroblasts, and endothelial cells to engineer full-thickness skin constructs. The AME enhanced cellular proliferation, migration, and angiogenic potential, while the composite hydrogel maintained printability and structural fidelity. Cultured constructs exhibited accelerated epidermal stratification and vascular network formation, demonstrating the synergistic effect of bioactive extracts and multi-cellular encapsulation for promoting skin regeneration.^86^ GelMA has been shown to work better than gelatine alone, and studies have demonstrated the efficiency of GelMA not only in promoting wound closure but also in producing HSEs in combination with other materials, such as hyaluronic acid methacryloyl (HA-MA).^87–89^ These characteristics have prompted GelMA to be one of the most promising materials for developing not only skin models, but also to mimic other tissue architectures.

Beyond collagen-derived systems, silk-based biomaterials introduce a distinct class of protein hydrogels with superior mechanical robustness and slower degradation rates.^90^ While it lacks native RGD motifs, it offers a unique combination of tensile strength and biocompatibility, making it particularly suitable for applications requiring durable scaffolds, such as dermal regeneration.^91,92^ In bioprinting, silk is often used as a base or reinforcing material, sometimes blended with gelatine, collagen, or bioactive peptides to improve cellular response.^93,94^ It can also be chemically modified with methacrylate or other functional groups to enhance crosslinking. Xu et al. developed a 3D-bioprinted skin patch composed of silk fibroin methacryloyl (SilMA) and GelMA. This combination provided enhanced mechanical stability, tunable degradation, and a bioactive environment supporting fibroblast and keratinocyte proliferation. In vitro and in vivo studies demonstrated accelerated wound closure and improved tissue remodelling when compared with the control, highlighting the synergistic potential of hybrid protein-based bioinks for cutaneous regeneration.^95^

Another naturally derived protein material, fibrinogen, has attracted significant attention for its dynamic mimicry of the wound-healing environment.^96^ Fibrinogen exists in fluid form in the bloodstream but polymerises into fibrin upon interaction with thrombin, forming a provisional ECM that supports angiogenesis, fibroblast proliferation, and keratinocyte migration.^97,98^ Fibrinogen-based bioinks are especially valuable in modelling inflamed or regenerating skin environments.^99^ It can promote vascularisation in constructs by supporting endothelial cell sprouting and network formation.^100^ However, the fast degradation and relatively weak mechanical properties may require blending with more stable materials like gelatine, agarose, or silk to extend construct longevity.^101^ Recent work by Martin-Piedra et al. evaluated nanostructured fibrin–agarose skin substitutes in burn patients over a time-course study. Histological analyses demonstrated progressive integration, vascularisation, and stratification of the epidermal and dermal layers. This work highlights the clinical potential of fibrin–agarose matrices as bioactive scaffolds for accelerated wound healing and tissue regeneration.^102^

In parallel to protein-based hydrogels, polysaccharide systems such as alginate have become relevant for their excellent printability and mechanical control. Alginate is widely used in skin engineering due to its exceptional biocompatibility with human cells, and the low cost and ease of obtaining.^103^ Alginate also exhibits strong shear-thinning properties and polymerises quickly, which influences the scaffold’s shape.^104^ However, it has certain limitations, such as the potential for impaired shape fidelity during bioprinting deposition due to its low viscosity or poor adhesion to cells due to the absence of cell adhesion sites.^105^ To overcome this, alginate is often blended with ECM proteins or functionalised with RGD peptides. This modification enhances its biological activity while preserving its favourable printing characteristics.^106^ In skin bioprinting, alginate serves as a structural support when it is blended with other materials, such as fibrinogen, improving fibrinogen’s mechanical properties. Murali et al. developed a 3D-bioprinted full-thickness skin construct composed of alginate, gelatin, diethylaminoethyl cellulose, and fibrinogen, incorporating fibroblasts and keratinocytes in a rat model. The engineered construct promoted accelerated re-epithelialisation, enhanced neovascularisation, and well-organised collagen deposition compared to controls, demonstrating the synergistic effect of combining alginate, gelatine, and fibrinogen to support cellular proliferation, ECM deposition, and tissue regeneration.^107^ Although less biomimetic compared to protein-based hydrogels, its mechanical integrity and mild gelation conditions make it useful for maintaining complex shapes and printing layered skin architectures.

While protein-based and polysaccharide-derived inks have advanced printability and mechanical control, they still fall short of fully replicating the native extracellular milieu. To address these limitations, recent advances have turned towards ECM–derived materials.^108,109^ Several studies have used dECM-based bioinks, with the expectation that these bioinks would enhance cell proliferation and differentiation by providing essential biomolecules, including growth factors.^110–113^ Moreover, the encapsulation of cells such as endothelial progenitor cells (EPCs) and adipose-derived stem cells (ADSCs) in dECM has been demonstrated to promote neovascularisation, re-epithelialisation, and wound closure in vivo.^114^ While native dECM offers many biologically relevant components, its poor mechanical stability and weak printability limit its standalone use in biofabrication. Strategies such as methacrylation of dECM (dECM-MA) allow for tuneable polymerisation and mechanical reinforcement while preserving the ECM’s native bioactivity.^115,116^ Yu et al. developed a dECM-MA derived from porcine skin. The dECM-MA preserved key collagenous and matricellular components, providing a highly bioactive microenvironment that supported fibroblast proliferation, keratinocyte migration, and balanced ECM remodelling. In vivo, the hydrogel accelerated wound closure, enhanced angiogenesis, and promoted organised collagen deposition, leading to improved tissue architecture and reduced scarring. The study reinforces the translational potential of dECM-derived hydrogels as biologically faithful scaffolds for next-generation skin repair strategies.^117^ In this way, methacrylation bridges the gap between natural biomimicry and engineering control, transforming dECM into a more versatile and reproducible platform for HSE development.

Collectively, these biomaterials define a spectrum between intrinsic biological fidelity and engineered functionality. By carefully tuning stiffness, viscoelasticity, and other mechanical parameters, these composite scaffolds can better mimic the native mechanobiology of skin, influencing cell behaviour, tissue maturation, and wound healing responses. Hence, optimal biomaterials for HSEs will likely depend on composite strategies that combine their strengths while mitigating their respective limitations.

### 3D Bioprinting methods

**Figure 2.**
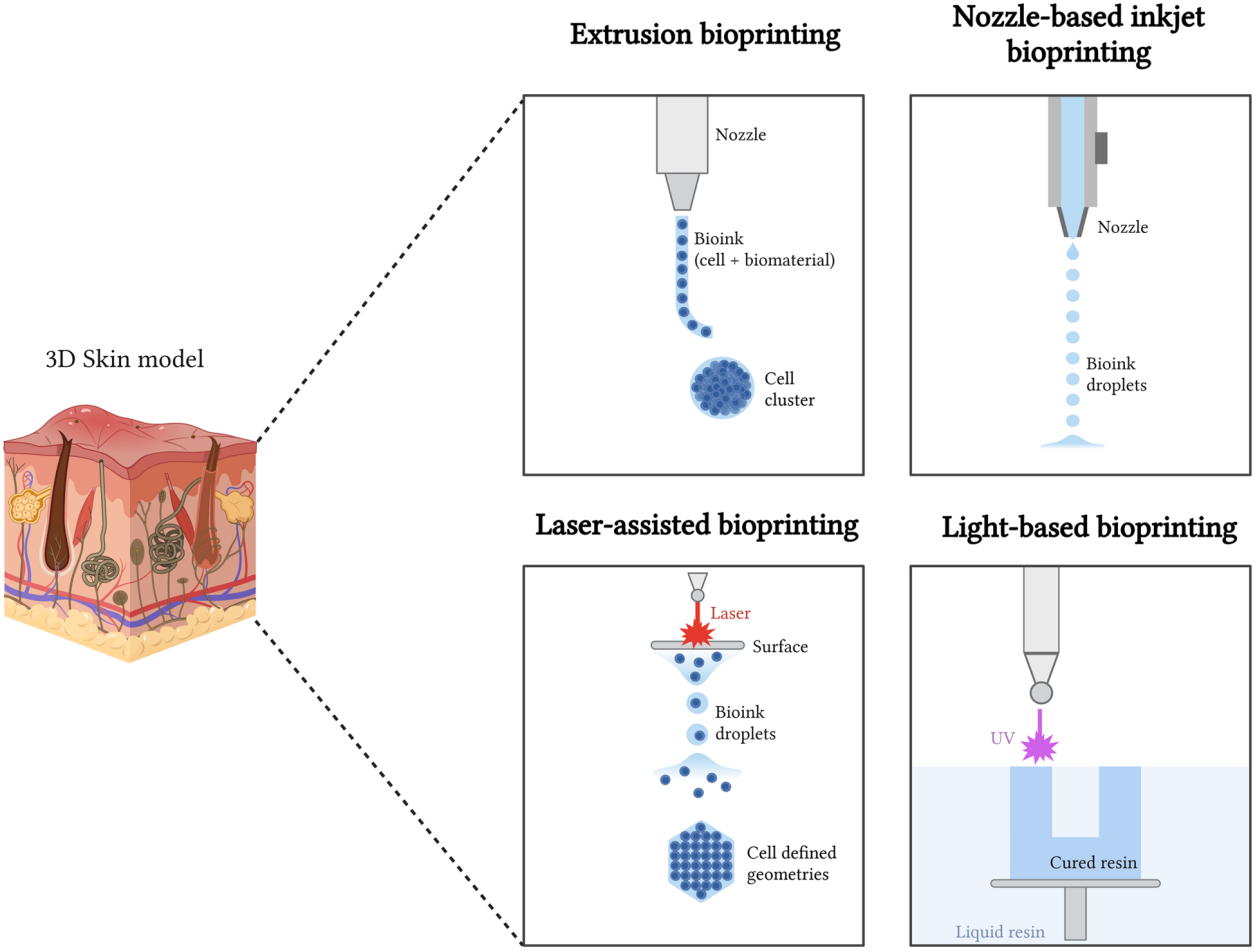
Overview of common bioprinting technologies used in skin model development. Extrusion-based bioprinting enables the deposition of viscous bioinks to build multi-layered skin structures. Inkjet bioprinting enables high-resolution droplet-based deposition, making it suitable for cell patterning. Laser-assisted bioprinting offers precise cell placement through laser-induced forward transfer. Light-based bioprinting utilises photo-crosslinkable bioinks to fabricate complex, high-resolution structures through layer-by-layer photopolymerisation. Each technique presents distinct advantages in terms of resolution, speed, cell viability, and material compatibility for engineering functional human skin equivalents. Created with Biorender.

Together with biomaterials, biofabrication techniques have evolved considerably through the years. Biofabrication refers to the automated generation of biologically functional products with structural organisation from living cells, bioactive molecules, biomaterials, and cell aggregates, through bioprinting or other techniques and subsequent tissue maturation processes.^118^ Unlike traditional methods, where cells are seeded onto pre-formed scaffolds, bioprinting allows for the direct placement of multiple cell types and biomaterials in predefined patterns.^119^ Recent advances in bioprinting technologies have enabled the development of multilayered skin constructs with high spatial resolution, allowing for the precise incorporation of multiple cell types, biomaterials, and biochemical gradients that closely emulate the native skin microenvironment.^120,121^ A key component of these approaches is the use of bioinks, composite formulations of cells and biomaterials, which serve as both the structural scaffold and the biological milieu in which cells proliferate, differentiate, and deposit their extracellular matrix.^122^ There are four broad bioprinting approaches to produce HSEs. These are extrusion-based, nozzle-based jetting bioprinting, laser-assisted, and light-based bioprinting, such as digital light processing (DLP)^123,124^ (Figure 2).

#### Extrusion-based bioprinting

Extrusion bioprinting is the most widely used technique for fabricating skin constructs due to its ability to print high-viscosity bioinks and accommodate high cell densities. In this method, bioinks are dispensed through a nozzle using pneumatic or mechanical force to form continuous filaments.^125^ This approach is particularly suitable for printing dermal scaffolds composed of collagen, gelatine, or fibrin, and for depositing distinct layers such as dermis and epidermis. Lee et al. developed a bioprinted dual-layer skin construct that incorporates a vascularised dermis-epidermis layer on top of an adipose tissue base to mimic native skin architecture better. The adipose layer was created using 3T3-L1 adipocyte spheroids embedded in GelMA-coated PCL scaffolds, enhancing adipogenesis and cell viability. A vascularised skin layer composed of HUVECs and fibroblasts embedded in microgels was printed on top, leading to organised endothelial network formation. This fully bioprinted, multilayered model supports physiologically relevant studies of adipose–skin interactions, vascularisation, and wound healing, offering an improved in vitro platform for investigating complex skin disorders and regeneration processes.^126^ In another study, Andrade et al. bioprinted a robust human skin co-culture model using high-viscosity fibrin-based bioinks. Cell viability assays at 1, 10, and 20 days post-printing demonstrated significantly higher survival in co-culture constructs than in monoculture controls, with sustained proliferation and no apparent competition between cell types. Rheological characterisation revealed that keratinocyte presence notably influenced the viscoelastic properties, storage and loss moduli, of the hydrogel, underscoring the importance of cell-specific bioink tuning. Immunocytochemical analysis further confirmed phenotypic maintenance (K5/K10 for keratinocytes; vimentin/FSP for fibroblasts) and indicated enhanced structural and functional integration. These findings highlight the critical role of cell–cell interactions in optimising bioink formulations and support the platform’s potential for creating viable, mechanically stable 3D skin constructs for tissue engineering applications.^127^

However, extrusion-based also has some limitations. The resolution is limited compared to other bioprinting techniques and the extrusion of high-viscosity bioinks needs elevated pressure, generating substantial shear stress at the nozzle interface that may compromise cellular integrity.^128,129^

#### Nozzle-based jetting bioprinting

There are several forms of jetting bioprinting, piezoelectric, thermal, and microvalve, which involve the dropwise deposition of small volumes of low-viscosity bioinks.^130^ This non-contact, high-speed method enables precise cell placement and is cost-effective for generating layered skin models.^131^ This technology has the potential to be used for epidermal layering. Lee et al. combined pneumatic microextrusion and inkjet modules to fabricate structured, tri-layered human skin equivalents in a single-step process. The extrusion system deposited a dermal compartment composed of collagen and primary human dermal fibroblasts, reinforced by an embedded polycaprolactone (PCL) mesh to prevent contraction during maturation. Concurrently, the inkjet module enabled uniform seeding of keratinocytes onto the dermal layer, facilitating the formation of a stratified epidermis under ALI culture. The resulting construct maintained structural fidelity and exhibited a stabilised fibroblast-populated dermis with fully stratified epidermal layers after 14 days. This integrated approach demonstrates precise spatial control over multi-cellular architecture and resource-efficient fabrication, highlighting significant progress towards scalable, reproducible human skin models.^132^ They then validated and used this model to examine the effects of *Aronia melanocarpa* extract on human skin conditions.^133^

Moreover, this technology could be used together with other bioprinting strategies. Recently, Jiao et al. developed a verteporfin-loaded bilayer skin substitute using a hybrid bioprinting approach that combines extrusion printing for the dermal layer, incorporating fibroblasts and endothelial cells, and inkjet printing for the epidermal layer with keratinocytes and melanocytes. In comparison with the control wound, the bioprinted skin enhanced vascularisation by 123%, supported multilayered epidermal regeneration with pigmentation, and improved collagen and fibronectin deposition, collectively accelerating wound healing and reducing scarring. This study highlights the potential of combining extrusion and inkjet bioprinting to fabricate functional, complex skin models for regenerative medicine.^134^ Despite its high resolution, low cost, and ability to deposit cells and biomaterials with precision, inkjet bioprinting remains limited by low cell density, restricted bioink viscosity, the potential for clogging, and low mechanical strength of constructs.^135,136^

#### Laser-assisted bioprinting

Laser-assisted bioprinting (LAB) offers exceptionally high resolution and cell viability by using a focused laser pulse to propel droplets of bioink from a donor slide onto a substrate. This method allows for the precise spatial arrangement of multiple cell types, making it well-suited for patterning complex skin features such as vasculature, pigmentation, and appendages.^137^ Salvadori et al. presented a high-resolution laser-based method to fabricate controlled microvascular patterns in collagen-based hydrogels for organ-on-chip applications. Using infrared laser pulses, the team precisely ablated microchannels with diameters ranging from 10 to 50 µm, mimicking physiological capillary dimensions without requiring sacrificial materials or altering the bulk hydrogel architecture. Endothelial cells seeded into these channels exhibited high viability and formed confluent, lumenised linings, maintaining barrier function under continuous perfusion for up to 10 days. The platform was successfully integrated into a microfluidic chip, advancing vascularised tissue models and the physiological relevance of organ-on-chip systems.^138^ While LAB offers excellent cell viability, high resolution, and nozzle-free precision, its clinical translation is hindered by high operational costs, complex setup requirements, and limited scalability, making it less accessible for large-scale fabrication of functional skin constructs.

#### Light-based systems

Light-based bioprinting techniques, such as DLP, use photopolymerisable inks to construct structures with micron-scale resolution. These materials undergo light-induced crosslinking, a chemical process in which photo-reactive groups form covalent bonds upon exposure to specific wavelengths of light. This creates a stable polymer network, enhancing the material’s mechanical strength, structural integrity, and long-term stability.^139^ These techniques allow rapid crosslinking and the fabrication of geometrically complex constructs.^140,141^ For skin models, DLP can produce vascular-like channels and porous dermal matrices with high fidelity.^142^

A recent study by Choi et al. developed a full-thickness artificial skin model using DLP with a bioink composed of methacrylated silk fibroin and Gel-MA. The optimised formulation exhibited favourable rheological and mechanical properties, high printing fidelity, and sustained cell viability. The engineered constructs supported stratification and differentiation of keratinocytes, proliferation of dermal fibroblasts, and the formation of endothelial structures over a 4-week ALI culture. Furthermore, the incorporation of a full-thickness wound model enabled quantitative assessment of regenerative responses, demonstrating that exogenous epidermal growth factor (EGF) significantly accelerated re-epithelialisation and neovascularisation, as evidenced by enhanced expression of CK13, FGF, and CD31. These findings underscore the utility of DLP-printed Silk-GMA and Gel-MA based platforms as advanced in vitro models for studying wound healing dynamics and the potential screening of therapeutic agents.^143^ Although light-based bioprinting technologies offer high resolution and rapid fabrication of complex architectures, their broader application is limited by constraints in bioink formulation, potential phototoxicity, and the challenge of integrating living cells without compromising viability.^144,145^

While each bioprinting modality offers unique advantages and faces distinct limitations, their continued evolution has laid the groundwork for fabricating increasingly complex, multilayered skin constructs. To contextualise their translational relevance, it is essential to compare them with conventional approaches that have historically shaped the field.

## Current state of the art in skin modelling

**Figure 3.**
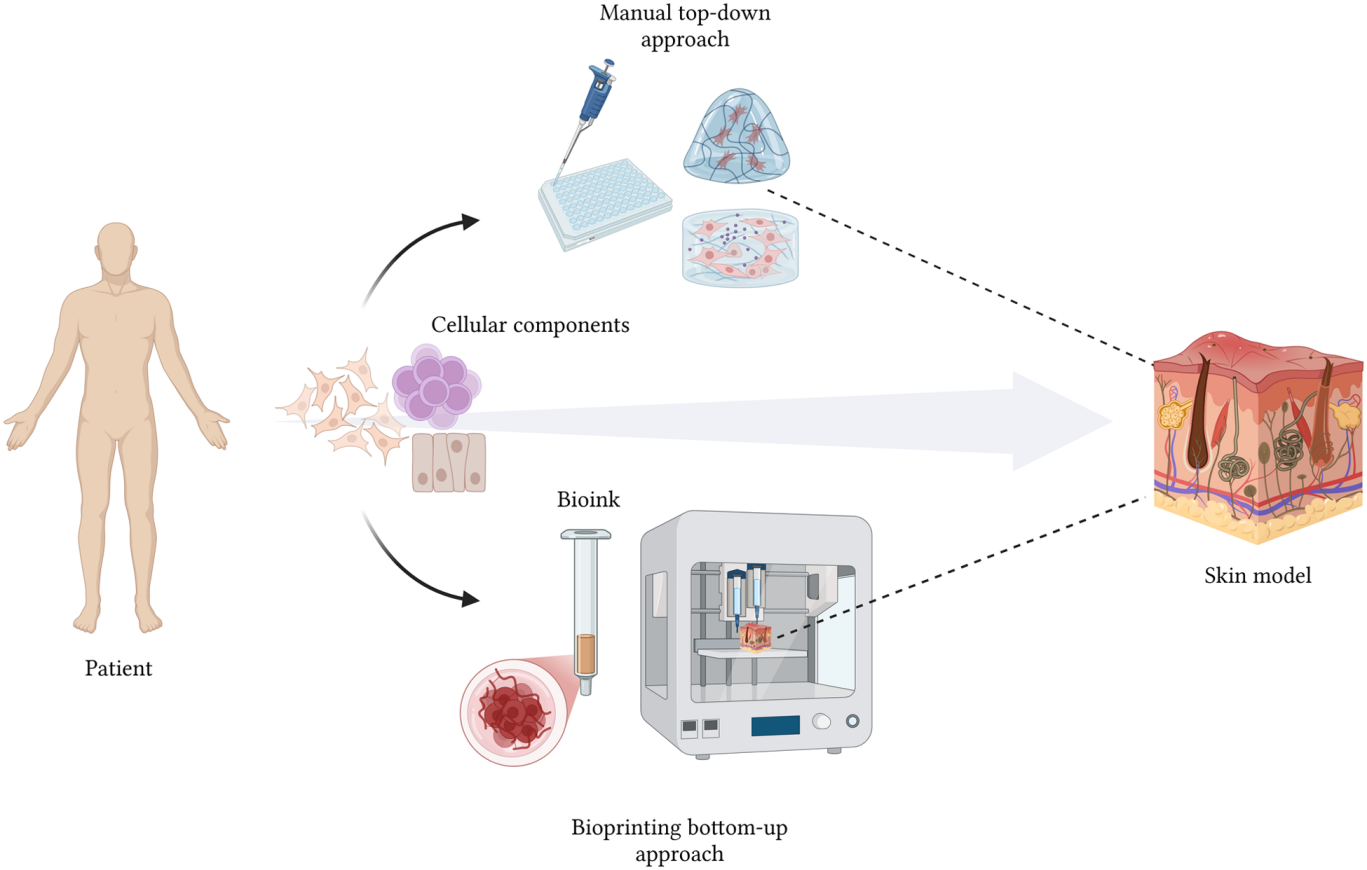
Schematic representation of skin structure modelling through top-down and bottom-up approaches. Although both techniques allow the development of a skin model, the use of bioprinting enables the precise incorporation of multiple cellular components, functional skin structures, and biomaterials to closely mimic the native architecture and complexity of human skin. Created with Biorender.

The growing demand for physiologically relevant skin models has driven innovation across materials science, tissue engineering, and biofabrication. From early, simplistic constructs to today’s multilayered, highly organised skin equivalents, the field has steadily progressed towards systems that more accurately replicate native skin architecture and function. Current strategies range from top-down approaches, where cells are manually seeded onto scaffolds, to bottom-up biofabrication techniques that build skin equivalents through the precise assembly of cellular and matrix components (Figure 3). This evolution reflects not only technological advances in scaffold design and cell culture but also the increasing need for reproducibility, standardisation, and translational relevance across both academic and industrial settings.

Commercially available skin models have largely been developed using conventional top-down fabrication approaches. While these methods have laid the groundwork for in vitro skin research and preclinical testing, they are inherently limited by non-uniform cell distribution, batch variability, and limited architectural complexity.^146^ In contrast, bottom-up biofabrication platforms offer greater design flexibility, automation, and reproducibility, marking a substantial leap towards high-resolution, complex, and functional skin constructs.^147^ Together, these complementary strategies define the current state of the art in engineered skin models, as summarised in Table 3

**Table 3.** Current in vitro HSE available from traditional tissue engineering to bioprinting.

Manual skin engineering method			
Cell types	Finding	Material	Reference
NHDF, HEK, hiNSCs	The innervated, full-thickness HSE demonstrated use for long-term culture for up to 6 weeks	Silk	Vidal et al.^147^
NHDF, HEK, T-cells	Impact of the infiltration of activated T cells on the development of psoriasis, finding moderate activation of STAT1 pathways	Self-assembly method	Lorthois et al.^148^
NHDF, HEK, T-cells	Incorporation of patient-derived T cells to analyse immune responses of the HSE, T-cell migration, and drug responsiveness	Type 1 Collagen matrix (animal not specified)	Shin et al.^149^
NHDF, NFF, DFUF, NHK, Monocytes	Developed an ECM produced by fibroblasts that retains the epithelial-mesenchymal crosstalk and supports macrophage survival	Bovine Type 1 Collagen	Smith et al.^150^
NHDF, Melanocytes, HEK, melanoma cells, Monocytes	Developing an early model of melanoma that supports the differentiation to M2 macrophages due to IL-10 released.	Rat tail collagen and fibrinogen	Michielon et al.^151^
DFUF, NFF, Monocytes, HEK	Monocytes differentiate into macrophages either directly into the HSE or polarised before adding	Bovine Type 1 Collagen	Smith et al.^152^
NHDF, HEK, T-cells	Shows the best isolation and activation of T-cells and how the interaction of these cells with pathological epithelial cells promotes the inflammatory environment found in psoriasis.	Self-assembly method	Rioux et al.^153^
NHDF, HEK, Dermal single-cell suspension (endothelial and immune cells)	They obtained the first tissue-engineered skin model that is fully autologous, vascularised, and immunocompetent.	Self-Assembly Method and Type 1 Bovine Collagen	Attiogbe et al.^154^
NHDF, Macrophages	A 3D model that can analyse tissue changes due to bacteria in the short term (3 days)	Collagen-based matrix from pig intestine	Murkar et al.^155^
NHDF, HEK, T-cells	Developed a psoriasis and atopic dermatitis model that presents several clinical features of inflammatory diseases. This could be useful as a platform to study inflammatory skin diseases	Self-assembly method and Collagen-matrix type 1 (No animal specified)	Scheurer et al.^156^
NHDF, HEK, THP-1	Analyse how Thp-1 monocytes react when exposed to UV light. They found that Thp-1 monocytes polarise to different types of macrophages	Fibrinogen	Phuphanitcharoenkun et al.^157^
Immunocompetent bioprinted skin models			
Cell types	Finding	Material	Reference
NHDF, HEK	Develop a new bioink that facilitates physiological processes such as ECM synthesis, macrophage polarisation, and angiogenesis	Alginate-Gelatine and 5% PRP (platelet-rich plasma) ink	Zhao et al.^158^
NHDF, HUVEC, HEK, Macrophages	Developing a matured immunocompetent skin-like printed model, enabling the evaluation of inflammatory responses following the topical application of various substances	SilkMA, GelMA and PRP	Bhar et al.^159^
Recent bioprinted skin models			
Cell types	Finding	Material	Reference
NHDF, HEK, ADSCs	Production of a three-layered structure containing epidermis, dermis, and hypodermis by suspended additive manufacturing	Type 1 Bovine Collagen and Pectin	Moakes et al.^160^
CSCs, MM, NHDF, HEK, HUVECs	The first malignant melanoma model was developed. This model also allows the recreation of a similar human structure and composition when implanted in mice	Type 1 Rat Tail Collagen	Andrés et al.^161^
NHDF, HEK	Although lacking other cell types showed a similar native skin architecture with a novel bioink.	Fibrinogen-Alginate	Cavallo et al.^76^
NHDF, HEK	Bioprinting strategy to model rete ridges in the skin	Collagen-Fibrinogen	Chae et al.^162^
NHDF, HEK, and hMSCs for adipocytes differentiation	Bioprinted skin model with three layers (epidermis, dermis, and hypodermis) to analyse the impact of hypodermis in skin equivalents.	Type 1 Rat Tail Collagen	Avelino et al.^163^
NHDF, HEK, Melanocytes	First bioprinted skin model using plant-derived recombinant human collagen. The model includes epidermal stratification and melanin production, expressing relevant markers such as involucrin and cytokeratin 14	Recombinant human type I collagen	Gudapati et al.^164^

There has been a significant growth in the development of 3D bioprinted skin models. Some studies are trying to incorporate nerves into the model. Muller et al. presented a pioneering model of human innervated skin by incorporating induced pluripotent stem cells (iPSCs)-derived sensory neurons and Schwann cells into a 3D collagen-based scaffold. The authors successfully differentiated these cells from the same donor line and demonstrated functional integration, with neurons extending neurites into the epidermis and releasing neuropeptides upon stimulation. This model marks an important step towards mimicking human neurocutaneous physiology, enabling the study of neuroinflammatory mechanisms in skin disorders. However, the construct lacks vascular and immune components, as well as full epidermal stratification, limiting its complexity and in vivo relevance.^165^ More recently, Rousi et al. developed a fully human, innervated 3D skin model incorporating iPSC-derived sensory neurons into a tissue-engineered construct composed of primary keratinocytes and fibroblasts embedded in a fibrin-based hydrogel scaffold reinforced with aligned electrospun fibres. The model supports axonal outgrowth into the epidermis and exhibits stratification with relevant epidermal markers, offering a physiologically relevant system to study neurocutaneous interactions. Functional assays confirm the responsiveness of neurons to noxious stimuli, highlighting the platform’s utility for dermatotoxicity and sensitisation studies. Importantly, the use of exclusively human-derived cells, without reliance on animal models or explanted nerves, marks a significant advancement towards scalable, standardised in vitro systems for studying sensory function in skin.^166^ Despite partial epidermal maturation and the absence of immune or vascular components, this model represents a critical step forward in bridging skin biology with neural input, with implications for pain, inflammation, and skin–nerve crosstalk research. However, none of these models have yet been developed using bioprinting, highlighting this as an emerging and underexplored niche with significant potential for future research. Incorporating the hypodermis into bioprinted HSEs is crucial, as it contributes to endocrine signalling, immune regulation, and mechanical support. Its inclusion significantly enhances the physiological relevance and regenerative capacity of engineered skin constructs. Lee et al. introduces a compelling strategy for the integration of functional adipose tissue within bioprinted skin constructs to enhance regenerative outcomes. Using a hybrid bioink composed of adipose-derived decellularised extracellular matrix (adECM) and alginate, a modular bioprinting platform that enables spatially defined assembly of endocrine adipose units was established. By optimising unit dimensions and interspacing based on diffusion modelling, the engineered constructs promoted adipogenesis and paracrine signalling, while maintaining structural fidelity. In vitro, the system enhanced keratinocyte migration and upregulated genes critical for skin remodelling, including *COL1A1*, *MMP2*, and *ITGB1*. Importantly, in a full-thickness murine wound model, the adipose-integrated constructs accelerated re-epithelialisation, neovascularisation, and extracellular matrix organisation.^167^ This work underscores the therapeutic potential of bioprinted endocrine-active adipose units and represents a significant step towards the fabrication of physiologically relevant skin equivalents for translational wound healing applications.

Other approaches have focused on modelling systemic disease states such as diabetes, which profoundly affect skin physiology and wound repair. Smith et al. developed three types of dermal matrices comprising human foreskin, nondiabetic adult-foot, and diabetic foot-ulcer fibroblasts. The dermal matrix was stabilised for over 7 days, and the results demonstrated that fibroblasts isolated from diabetic foot ulcers deposited an endogenous ECM de novo, which formed an environment similar to the one from which they were originally harvested. Moreover, they co-cultured monocytes in the model and differentiated them into the M1 phenotype.^168^ This study managed to obtain an M1 phenotype through monocyte differentiation, which is promising and can be a way of incorporating an immature immune system. Integrating in the model diabetic derived cells can be a promising method to develop HSEs that contain hallmarks of complex wounds and immune response. Maione et al. built a diabetic model based on a patient with a diabetic foot ulcer-derived fibroblasts encapsulated in a collagen type I matrix. The model showed several hallmarks of chronic ulcers, including impaired angiogenesis, re-epithelialisation, and ECM deposition.^169^ However, there are still some limitations, as these studies did not compare the poor wound-healing capabilities, which constitute the major hallmark of complex wounds. Some models have tried this through bioprinting. Kim et al. developed a 3D bioprinted diabetic-skin model representing the pathological features of diabetes, which demonstrated impaired wound-healing ability when compared to the control. They engineered a construct from diabetic fibroblasts from the patient, seeded together with HUVECs in a skin-derived decellularised ECM. Following incubationkeratinocytes were then distributed using an inkjet module on top of the ECM. The study confirmed that the interaction between diabetic fibroblasts and keratinocytes leads to a stratified, mature epidermis. Moreover, the impaired wound-healing ability was successfully achieved when compared to the control. Furthermore, the hypodermal compartment, which included a perfusable vascular channel, was incorporated beneath the skin model. This diabetic-skin model showed applicability for drug screening when diabetic characteristics were improved via treatment with metformin, a well-known drug for treating diabetes, through the vascular channel.^170^ The incorporation of multiple layers and diverse cell types in recent constructs has not only enhanced physiological relevance but also positioned these models as promising candidates for in vivo wound healing applications.

### Skin models for in vivo wound healing applications

Building on this progress, recent efforts have focused on translating engineered skin models into preclinical and clinical settings. These in vivo applications highlight the potential of bioengineered skin to support tissue regeneration, modulate immune responses, and accelerate wound closure in challenging environments. One notable example comes from Jorgensen et al., who developed a tri-layered bioprinted skin containing six human cell types. When transplanted into mice, this construct achieved complete wound closure within 14 days and displayed organised collagen remodelling with basket-weave architecture by day 90. They also conducted a porcine pilot study with the same construct showing accelerated wound closure, reduced contraction, and favourable expression of ECM-related genes, including a TGF-β1/β3 ratio suggestive of scarless healing.^171^ Recent efforts have also focused on optimising the bioink environment to promote endogenous repair. A promising approach combined decellularised adipose ECM with GelMA and HA-MA hydrogels, embedded with ADSCs. This construct enhanced re-epithelialisation, neovascularisation, and collagen deposition in murine full-thickness wounds, while significantly reducing scar formation and inflammation.^172^ The addition of endothelial progenitor cells (EPCs) further improved wound closure dynamics in diabetic models, underlining the synergistic potential of ADSC-EPC co-delivery in a supportive ECM-based scaffold.^173^ Composite materials have also been explored to improve mechanical strength and mimic native ECM components. Zhang et al. developed a microfragmented adipose-derived matrix within a bioprinted scaffold, which enhanced epithelial regeneration and dermal integration in full-thickness wounds.^174^ Other strategies, such as inkjet bioprinting, have demonstrated relevance in in vivo wound models. An inkjet-printed dermal equivalent composed of fibroblasts, keratinocytes, and endothelial cells deposited within a collagen matrix resulted in reduced wound contraction, enhanced angiogenesis, and the development of distinct dermal and epidermal layers in nude mouse models.^175^ Despite these promising developments, several limitations remain. Most studies rely on small animal models that lack the full immunological and mechanical complexity of human skin. Collectively, these studies demonstrate the rapidly maturing potential of bioprinted HSEs in wound healing. Their ability to reproduce native architecture, integrate multiple cell types, and support functional regeneration positions them as strong candidates for translational applications.

Another promising technique that will be briefly mentioned in this review is in situ bioprinting. This technology has emerged as a next-generation strategy in skin tissue engineering, enabling direct deposition of bioinks onto wound sites to fabricate constructs that conform to patient-specific geometries. Recent advances in handheld and robotic in situ bioprinting systems have demonstrated remarkable flexibility and therapeutic potential. Wang et al. developed a programmable, smartphone-controlled handheld bioprinter that was shown to deposit multilayered bioinks with high precision and more than 79% cell viability, significantly accelerating wound closure in a rat model.^176^ Zhao et al. developed an adaptive multi-degree-of-freedom robotic system for in situ bioprinting that enables precise deposition of cell-laden bioinks onto irregular skin wounds in mice. The platform successfully promoted regeneration of full-thickness skin, including hair follicles and vascular networks, features rarely achieved in prior in situ models. This study highlights the potential of advanced robotic bioprinting to fabricate complex, functional skin tissues directly on wounds, moving closer to clinical translation^177^ especially as the adoption of robotic platforms in surgery is increasing. Bioink design is crucial for in situ application, requiring rapid crosslinking, mechanical stability, and high biocompatibility under physiological conditions. Zhou et al. designed a new system, termed the SkinPen, using GelMA combined with copper-doped bioactive glass nanoparticles (Cu-BGn) and dual UV/ultrasound-assisted gelation, achieving strong adhesion, antimicrobial activity, and angiogenic promotion in vivo.^178^ Despite this progress, major limitations remain. These include challenges in maintaining print fidelity on moist, irregular, or mobile tissue surfaces, limited resolution compared to benchtop bioprinters, and the need for scalable, biocompatible crosslinking methods.^179^

Despite notable advancements in 3D bioprinting of skin, current models remain limited in their capacity to fully recapitulate the structural, functional, and immunological complexity of native human skin. Most bioprinted constructs rely on simplified dermal–epidermal architectures, often lacking key appendages such as hair follicles, sweat glands, and a functional vascular network. Furthermore, the lack of perfusable microvasculature limits nutrient diffusion and restricts model viability beyond several days. Moreover, most in vitro skin constructs fail to capture the dynamic immune responses that shape wound healing, making them insufficient for modelling complex non-healing wounds.

### How can the current HSEs platforms be improved?

**Figure 4.**
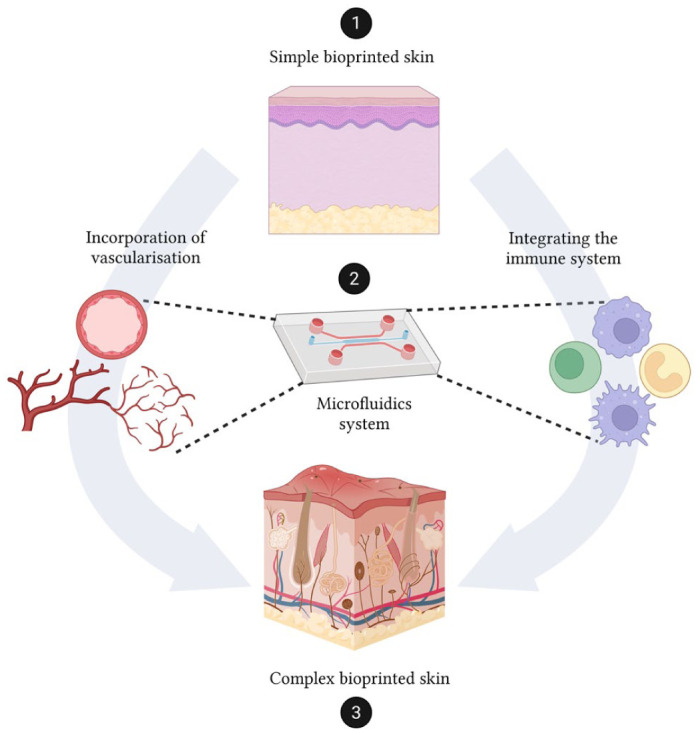
Schematic representation of the stepwise development and improvement of current HSEs platforms. (1) A simplified skin construct is first generated. (2) Integration of vascular and immune components is achieved using a microfluidic platform, enabling dynamic perfusion and immune cell interactions. (3) The resulting system recapitulates key structural and functional hallmarks of native skin, representing a complex, immunocompetent, and perfused skin model suitable for advanced studies in physiology, disease modelling, and therapeutic screening. Created with Biorender.

There has been a huge growth in the development of 3D printed models but, despite these advances, current constructs still face challenges in fully replicating the structural, cellular, and functional complexity of native skin. Continued progress will depend on integrating vascularisation, immune competence, and appendage regeneration, as well as developing standardised protocols that bridge the gap between experimental models and clinical translation (Figure 4).

To better mimic native skin physiology, recent studies have focused on incorporating both vascular and immune components into HSEs. Vascularisation not only enhances nutrient and oxygen delivery but also supports immune cell trafficking and paracrine signalling critical for tissue homoeostasis and repair.^180^ Simultaneously, the inclusion of immune components such as macrophages enables more accurate modelling of inflammation, host–pathogen interactions, and wound healing responses. The synergistic integration of these systems is paving the way for more functional skin models.

#### Vascularisation

One of the key limitations of current HSE models is the lack of a functional vascular network. As mentioned before, in native skin, vascularisation is essential for oxygen and nutrient delivery, waste clearance, immune surveillance, and coordination of repair processes, functions that become critical in thick or multilayered constructs.^181^ To address this, recent efforts have focused on integrating vascular structures using advanced biofabrication techniques, aiming to replicate not only the architecture but also the dynamic perfusion and signalling of native dermal vasculature. Enhancing vascularisation represents a pivotal step towards developing HSEs capable of sustained long-term viability, integration, and functional performance in both in vitro and in vivo contexts.^182^ To improve the formation of vascular trees for organ modelling, a novel study by Sexton et al. presents a model-driven design platform that drastically accelerates the creation of organ-scale synthetic vasculature. This novelty could address a critical barrier in bioprinting regarding vasculature and perfusable tissues. They optimised algorithms to generate vascular trees up to 230-fold faster than traditional methods, support arbitrarily complex geometries via localised implicit functions, and seamlessly connect vessels into watertight networks suitable for computational fluid dynamics and fabrication. The platform was validated across over 200 engineered and anatomical models, ranging from cubes and annuli to bi-ventricular hearts and brain, demonstrating robust haemodynamic performance in silico. The generated vasculature was successfully 3D printed using the FRESH printing approach, and perfusion within bioreactors improved cell viability in tissue constructs.^183^ This offers a scalable solution for designing and fabricating perfusable vascular networks, enabling the biomanufacturing of larger and more physiologically relevant tissues, including skin.

Recent work has begun to build vascular structures into 3D skin models through the use of microfluidic channels.^184,185^ Maggiotto et al. developed a novel 3D bioprinted, vascularised skin-on-chip model designed for drug testing and wound healing applications. Using a dual-bioink approach, the authors printed a dermal and epidermal layer of GelMA with sacrificial Pluronic F127 to create perfusable vascular channels that were later endothelialised with HUVECs and fibroblasts . The construct was integrated into a perfusion bioreactor, enabling continuous flow, which supported nutrient diffusion, validated via computational modelling and fluorescence assays. The printed tissue featured stratified keratinocytes, a proliferative dermal layer with fibroblast activity, and functional vascular structures. Notably, the model demonstrated complete re-epithelialisation over 14 days, aligning more closely with human skin biopsies than 2D cultures.^186^ Additionally, perfusion significantly reduced cytotoxicity compared to static cultures. In another study, Sun et al. managed to perfuse neutrophils to analyse their reaction against Herpes Simplex Virus (HSV). This vascularised 3D system replicates essential structural and functional features of human skin, including blood vessel-like microvasculature that supports nutrient uptake, immune responses, and drug delivery. The study demonstrated that free-flowing neutrophils exhibited key immune behaviours in response to HSV infection, including slowing, rolling along the vessel wall, and migrating through the endothelial layer. These findings highlight the model’s potential for studying infections, immune interactions, and therapeutic interventions in a physiologically relevant environment.^187^ However, they developed a skin-on-chip platform that increases the complexity of the model and still has several limitations, including incomplete immune system integration, often focusing primarily on neutrophil responses while lacking the full complexity of adaptive immunity. Long-term viability remains a concern due to limitations in nutrient diffusion and waste accumulation in the model.

Advances in biofabrication, biomaterials, and microfluidic systems have made significant improvements in mimicking native microvasculature.^188,189^ However, achieving a fully functional, perfusable network that supports long-term tissue survival and immune cell circulation is still a challenge. The vascular system is essential in mediating inflammatory responses, as it is a route for immune cells to travel between lymphoid organs and peripheral tissues, especially to wounds,^190^ where immune cells clean the wound and fight against pathogens.^191^ Hence, introducing vascularisation into tissue-engineered skin models remains a critical challenge, yet it is essential for enhancing cell viability, nutrient exchange, and immune system integration.

#### Integrating the immune system

Incorporating the immune system into an HSE is essential for accurately replicating natural skin function, wound healing, and disease responses.^190^ The immune system plays a key role in regulating inflammation, tissue repair, and protection against pathogens. Models previously mentioned tried to incorporate macrophages, however, it is a simplistic representation of what the immune system is. By integrating immune cells, HSE becomes more physiologically relevant, making it a better alternative to animal models. This allows the production of immunocompetent models for drug testing, regenerative medicine, and personalised therapies, bridging the gap between in vitro experiments and real human responses. New techniques and co-culture methods are necessary to recreate the complexity of the immune system in this HSE.

**Figure 5.**
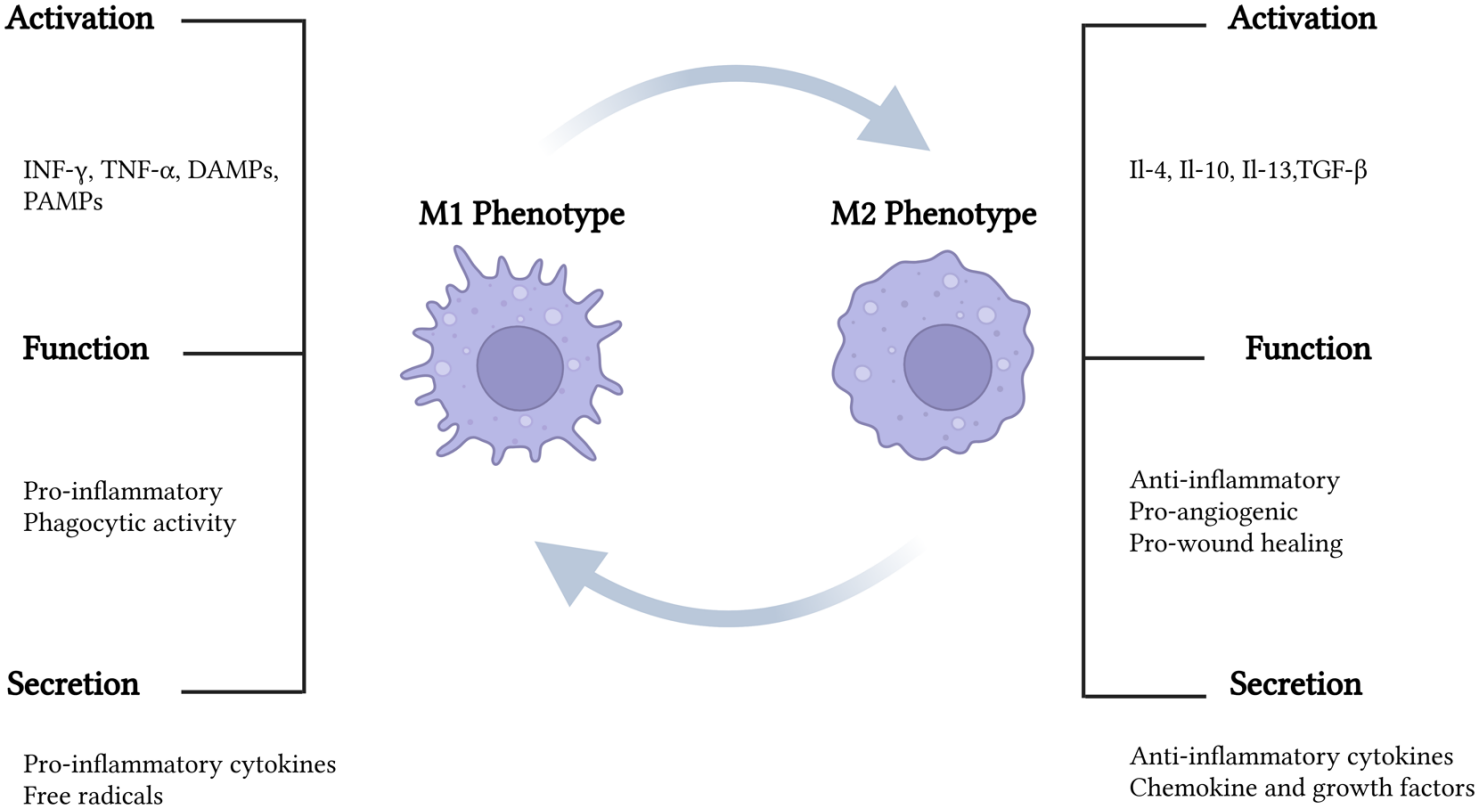
Overview of macrophage polarisation and phenotypic plasticity illustrating the classical in vitro classification of macrophages into pro-inflammatory (M1) and anti-inflammatory or pro-regenerative (M2) phenotypes. M1 macrophages are typically activated by IFN-γ and LPS, leading to the secretion of cytokines promoting inflammation and microbial clearance. In contrast, M2 macrophages are stimulated by interleukins IL-4 and IL-13 and are characterised by the production of anti-inflammatory cytokines and growth factors that support tissue repair and wound resolution. The bidirectional arrows represent macrophage plasticity, indicating that dysregulation of the wound environment or cytokine balance can drive reversible shifts between M1 and M2 phenotypes, influencing the outcome of skin regeneration and repair. Created with Biorender.

Macrophages are one of the key cells in the inflammation phase and, like fibroblasts, exhibit considerable plasticity and heterogeneity. These cells are derived from monocytes, and they are mainly found as M1 (pro-inflammatory) and M2 (anti-inflammatory) phenotypes. M1 macrophages are activated during infections or injury, producing pro-inflammatory cytokines (TNF-α, IL-6, IL-1β) and reactive oxygen species to clear pathogens, but prolonged activation can lead to chronic inflammation. In contrast, M2 macrophages support tissue repair, wound healing, and immune resolution by secreting anti-inflammatory cytokines such as IL-10 and TGF-β, promoting extracellular matrix remodelling and angiogenesis (Figure 5). In skin tissue engineering and wound healing, a controlled transition from M1 to M2 is essential for effective tissue regeneration.^192–194^ Incorporating macrophages in the HSE might be helpful to develop its own immune system.

A growing number of groups have begun integrating diverse immune cell populations, including Langerhans cells, dendritic cells, macrophages, and T cells, into 3D full-thickness skin equivalents, marking important progress towards the development of immunocompetent skin models that better replicate native skin physiology and immune responses.^195,196^ These models are typically constructed using transwell systems, collagen-based scaffolds, and primary human cells, which together enable the study of dynamic immune-dermal-epithelial interactions in a controlled environment.^197^ However, most of these models that are reviewed in the literature^198^ have been developed by other tissue engineering methods rather than bioprinting. In a couple of recent studies, immune cells were deposited in skin models, suggesting that these types of cells can be incorporated into in vitro skin models, but it has yet to be determined whether these types of approaches can replicate all the essential processes involved in the immune response to infection or skin inflammation.^199^ Hindle et al. used a dynamic bioreactor for immune modelling in bioprinted skin. They developed a microfluidic HSE with a vascular microchannel located in the dermal layer, enabling the delivery of circulating immune cells. The study demonstrates that stimulating keratinocyte inflammation promotes monocyte recruitment from the vascular channel into the epidermal layer, effectively replicating dynamic inflammatory immune responses within the skin.^200^ Although there are different ways to try to incorporate the immune system and immune features in the HSEs, it is still difficult to incorporate different types of immune cells in the model without compromising the viability of the other cells. Therefore, more research is needed to solve the current problems in terms of making the model more immunocompetent.

## Regulatory, translational, and ethical considerations in bioprinted skin models

Regulatory pathways for bioprinted products depend strongly on their intended use. Constructs designed for implantation or therapeutic regeneration are generally regulated under medical device and advanced therapy medicinal product (ATMP) frameworks, such as those defined by the U.S. Food and Drug Administration (FDA) and the European Medicines Agency (EMA).^201,202^ These frameworks require extensive preclinical validation of safety, efficacy, and manufacturing consistency, often following Good Manufacturing Practice (GMP) and ISO 10993 standards for biological evaluation of medical devices.^203,204^ In contrast, bioprinted skin models developed for research, drug testing, or toxicity screening are typically governed by regulations on in vitro test methods rather than those for implantable products. These models fall under the scope of organisations such as the Organization for Economic Co-operation and Development (OECD) and the European Centre for the Validation of Alternative Methods (ECVAM), which have established guidelines for RhE and full-thickness skin equivalents used in skin irritation and corrosion testing.^205^ The OECD Test Guidelines 431 and 439 define performance standards and validation requirements for such in vitro assays, ensuring reproducibility, sensitivity, and cross-laboratory comparability. However, as bioprinting technologies evolve towards complex, patient-specific skin equivalents that blur the line between in vitro models and therapeutic constructs, regulatory classification becomes increasingly challenging and calls for harmonised frameworks that bridge these domains.

In this context, one of the major hurdles in advancing HSEs towards regulatory acceptance is the lack of standardised bioprinting protocols and validation criteria. Parameters such as bioink composition, crosslinking conditions, cell density, printing resolution, and post-print culture protocols vary widely among studies, hampering reproducibility and interlaboratory comparison.^206,207^ A recent round-robin study demonstrated considerable variability in printed constructs across laboratories, underscoring the urgent need for consistent methodologies and standardised evaluation criteria.^208^ Establishing such standards is essential to move bioprinting from experimental setups to clinically compliant manufacturing workflows. National and international networks play a pivotal role here, promoting material characterisation frameworks and unified bioprinting standards that enhance reproducibility and accelerate clinical translation.

Recent progress has been made towards these goals. The NIST Special Publication 1500-23, authored by Babakhanova et al., identifies key standards relevant to tissue-engineered medical products. These include ISO 20391-1 and 20391-2, which address methodologies for accurate cell counting, and ASTM F2739 and F3504, which focus on evaluating cell viability and proliferation within scaffolds. The report also emphasises the development of reference materials, such as calibrated scaffolds and tissue-mimicking constructs, to provide benchmarking tools for consistent assessment of bioprinted tissues. Moreover, it highlights the need for standardised bioink characterisation and validation protocols, critical for ensuring cross-laboratory comparability and regulatory readiness of biofabricated products.^209^ Complementary initiatives, such as the ASTM F3659-24 guide for bioinks and the ISO Technical Committee 261 on additive manufacturing, are beginning to address these standardisation gaps by defining early frameworks for material characterisation, sterility, and printing precision.

While such technical and regulatory efforts aim to ensure the safety, reproducibility, and scalability of bioprinted constructs, they must advance alongside ethical governance as the field moves towards patient-specific applications. From a translational perspective, the use of patient-derived cells in bioprinting introduces complex ethical and regulatory challenges. Autologous cell sourcing offers the advantage of immune compatibility but simultaneously raises issues related to informed consent, data privacy, and ownership of biological materials. The emergence of iPSCs adds another layer of complexity, as their indefinite self-renewal and broad differentiation potential prompt questions about long-term storage, traceability, and potential misuse of patient-derived lines.^210,211^ Addressing these concerns requires transparent consent procedures, clear governance over cell provenance, and adherence to international bioethical standards to ensure the responsible integration of patient-specific, bioprinted skin constructs into regenerative medicine frameworks.

Ultimately, these ethical, regulatory, and standardisation dimensions are interdependent components of the translational pathway for bioprinted skin. Realising its full therapeutic potential demands not only scientific rigour but also alignment with clinical and industrial frameworks. Progress towards harmonised guidelines, validated reference bioinks, and GMP-compliant bioprinting workflows will be crucial to bridge laboratory research with clinical practice. Such efforts will accelerate the translation of bioprinted constructs from experimental prototypes to reproducible, safe, and effective HSEs for wound-healing research and other regenerative applications. Readers are encouraged to consult the Standards Coordinating Body portal for further information on ongoing efforts and available resources related to tissue engineering and regenerative medicine standards and regulations (https://portal.standardscoordinatingbody.org/).

## Future perspectives

### Bioprinting of skin organoids

Emerging technologies such as organoids hold promise for creating skin models that better capture physiological complexity. Organoids are 3D cultures derived from stem cells that are capable of mimicking the spatial structure and physiological characteristics of organs in vitro.^212^ Skin organoids, derived from pluripotent stem cells or induced progenitor populations, have demonstrated the capacity to self-organise into miniaturised, stratified skin with hair follicles, sweat glands, melanocytes, and vasculature, mimicking the complexity of native skin, overcoming the biological limitations of most of the current 3D bioprinted skin constructs.^213^ When combined with high-resolution 3D bioprinting, these organoid units could be spatially deposited into permissive, bioinstructive matrices to guide the maturation and integration of each component in situ. For example, Zhang et al. combined 3D bioprinted alginate–gelatine scaffolds designed for MSC–derived sweat gland induction with hair follicle organoids, creating an unprecedented in vitro model featuring both sweat glands and hair follicles.^214^ Similarly, hair follicle organoids can be embedded with dermal papilla–like aggregates in defined dermal niches to promote folliculogenesis post-printing.^215,216^ Vascularisation can also be accomplished with this tool. In a recent study, Zhang et al. developed a novel 3D bioprinting strategy that integrates human-derived skin organoids, comprising keratinocytes, fibroblasts, and endothelial cells, into a bioengineered construct capable of accelerating full-thickness wound healing. Using a hybrid gelatine–alginate bioink and dual-crosslinking approach, the authors fabricated highly organised skin organoid spheres with distinct epidermal and dermal architecture. When implanted into immunodeficient mice, these constructs promoted rapid re-epithelialisation, enhanced neovascularisation, and reduced inflammation compared to controls. The approach bridges the gap between organoid biology and bioprinting by enabling spatially controlled, scalable fabrication of skin tissues with functional regenerative capacity, marking a significant step towards clinically translatable, personalised skin grafts.^217^ However, their use, as bioprinted skin, remains constrained by the absence of vascular and immune components.^218^ Moving forward, the fusion of organoid biology with 3D bioprinting will be essential not just for structural fidelity, but for engineering functionally skin tissues that emulate the dynamic physiology and repair capabilities of native human skin.

### Integrating 4D bioprinting and machine learning for dynamic skin constructs

4D printing is an evolution of 3D bioprinting that incorporates time as a dynamic factor, enabling printed constructs to change shape, function, or behaviour in response to external stimuli such as temperature, pH, moisture, or light.^219,220^ Using 4D printing and “smart” materials, also known as stimuli-responsive or shape-morphing polymers, would be useful to emulate the constantly changing environment of the wound.^221–225^ These “smart” materials often include shape-memory polymers (SMPs) and stimuli-responsive hydrogels such as pNIPAM-based or alginate blends that can deform, swell, or stiffen on demand.^226^ This could be useful to create structures and integrate them in the HSEs, such as vascularisation. A recent study conducted by Zhang et al. demonstrated a 4D biofabrication strategy for self-folding vascular bifurcations, where anisotropic hydrogel bilayers undergo programmed deformation to create perfusable T-shaped lumens. This work elegantly integrates mechanical design and endothelialisation, offering a pathway towards dynamic vascular modules that could, in the future, support more complex tissue constructs. While the system remains primarily a geometric proof-of-concept, its principles may inform future biofabrication of vascularised skin equivalents, where morphogenetic self-assembly could complement bioprinting precision.^227^ More recently, Pramanick et al. introduced an embedded bioprinting platform enabling 4D shape-morphing tissues within yield-stress granular hydrogels to guide structural alignment and maturation of iPSC-derived cardiac constructs. While the study demonstrates the power of dynamic geometry in evolving tissue architecture and function, translation to skin and wound-healing models remains to be established, particularly given the distinct mechanical demands and vascular/immune interactions. However, this is a promising tool with a lot of potential to revolutionise the skin engineering field.^228^ By integrating these responsive inks into bioprinted constructs, researchers can create skin substitutes that morph over time for improved wound coverage, adapt to dynamic tissue environments, or release antimicrobials or growth factors in response to infection or inflammation.^229^ This technology will enable the fabrication of programmable, adaptive skin constructs, advancing beyond static 3D models towards functional, environment-responsive tissue therapies.

Machine learning (ML) is another tool that is increasingly being incorporated in bioprinting to enhance precision, reproducibility, and customisation in tissue engineering.^230,231^ By analysing large datasets from printing parameters, material properties, and biological outcomes, ML algorithms can predict optimal printing conditions, correct for deviations in real time, design complex tissue architectures with high fidelity, optimise bioink formulations, layer-by-layer deposition accuracy, and post-print cell behaviour, including proliferation and differentiation.^232,233^ In a recent study by Shin et al. ML algorithms were employed to dynamically adjust key parameters such as pressure, pulse amplitude, and nozzle frequency, enabling consistent droplet generation even under variations in bioink viscosity and cell density. This adaptive control system effectively minimised variability while maintaining high cell viability and structural uniformity across printed constructs. By integrating feedback loops and predictive modelling, the approach reduced the need for manual calibration and operator intervention, marking a shift towards autonomous, self-optimising bioprinting systems.^234^ Moreover, ML can help interpret imaging and omics, especially transcriptomics data from printed tissues, to guide iterative improvements in the biomaterial design, vascularisation strategies, and functional outcomes, bringing bioprinted tissues closer to physiological relevance.^235^ The integration of ML within the domain of bioprinting remains at an early stage, with only a limited number of studies available to date. Preliminary studies, comprised mostly of proof-of-concept demonstrations, are primarily focused on constructs other than skin. A study employed ML algorithms to test scaffold performance for skin tissue engineering, although it employed electrospun scaffolds rather than bioprinted ones.^236^ Similarly, artificial intelligence (AI) has been employed to improve in situ skin bioprinting since it is time-sensitive and requires real-time monitoring of the printing process. Jin et al. demonstrated the development of multiple designs of deep neural networks to detect real-time print anomalies, including discontinuity and deformity, with high accuracy.^237^ As ML becomes increasingly accessible and datasets grow, its use with bioprinting could significantly advance the development of personalised and functional engineered tissues.

## Conclusion

Over the past decades, skin model technologies have advanced significantly, from simple two-layer constructs to increasingly complex, multilayered systems. In this review, we have outlined the current state of the art in engineered human skin models and their emerging potential for translational and in vivo applications. While these platforms have shown promise in recapitulating aspects of skin architecture and function, they remain limited in their ability to fully mimic native skin physiology. Critical features such as immune competence, vascularisation, and innervation are still largely absent, impeding both mechanistic studies and clinical translation. Among these challenges, incorporating functional immune components remains particularly difficult. The immune system plays a central role in skin homoeostasis and wound repair, and its dysregulation underlies numerous skin disorders. Without immunocompetent models, our ability to study these processes and develop targeted therapies remains constrained.

Looking ahead, the convergence of materials science, organoid technologies, and machine learning is likely to drive the next generation of skin equivalents. The development of biomimetic bioinks, controlled delivery systems, and the incorporation of vascularised and immune-competent organoids will be critical for achieving functional integration and personalised applications. Moreover, machine learning will increasingly guide the design and optimisation of constructs, from bioink formulation to spatial patterning. To facilitate clinical translation, standardisation of fabrication protocols, quality control metrics, and regulatory frameworks will be essential. Ultimately, 3D bioprinting holds the potential not only to transform personalised wound care but also to serve as a powerful platform for disease modelling, drug testing, and regenerative medicine, bridging the gap between laboratory innovation and clinical practice.
